# Eruption of the Permanent First Premolar Associated with a Mandibular Keratocystic Odontogenic Tumor after Marsupialization in a 9-year-old Boy: A Case Report with 2 years of follow-up

**DOI:** 10.30476/DENTJODS.2020.85780.1152

**Published:** 2021-06

**Authors:** Nima Farshidfar, Mahya Agharokh, Hossein Daneste

**Affiliations:** 1 Student Research Committee, Shiraz University of Medical Sciences, Shiraz, Iran; 2 Dept. of Oral and Maxillofacial Surgery, School of Dentistry, Shiraz University of Medical Sciences, Shiraz, Iran

**Keywords:** Odontogenic Cyst, Odontogenic Keratocyst, Keratocystic Odontogenic Tumor, Marsupialization, Tooth Eruption

## Abstract

Amongst odontogenic cysts, keratocystic odontogenic tumor (KOT) is a benign intra-osseous lesion, characterized by corrugated parakeratinized uniform stratified squamous epithelium, with potential for aggressive behavior and high tendency to recur. There are multiple treatment modalities for this cyst. Some surgeons prefer the conservative treatments such as marsupialization while the others prefer invasive treatments such as radical resection. The aim of this study was to present a case of KOT involving the right mandibular premolar area with an impacted tooth in a 9-year-old boy treated by marsupialization. The treatment resulted in eruption of the mandibular first premolar, and no signs of recurrence were observed after two years. Marsupialization was found to be an effective treatment in inducing the eruption of mandibular premolar associated with KOT in preadolescents and can be a reliable procedure to reduce recurrence tendency of KOT

## Introduction

In 1956, the word odontogenic keratocyst (OKC) was originally named by Phillipsen until it was classified as keratocystic odontogenic tumor (KOT) by WHO in 2005 [ 1]. Without considering which term is used, there are three main reasons making this lesion so important: more growth potential than other odontogenic cysts, higher recurrence rate and conceivable relationship with nevoid basal cell carcinoma [ 2]. Besides, this odontogenic tumor is a benign intra-osseous, uni- or multicystic lesion which tends to be aggressive and infiltrative [ 3].

KOTs are more prevalently seen in the posterior region of the mandible than in maxilla [ 4]. This lesion usually manifests during the second, third or fourth decades of life with a slightly higher occurrence amongst male [ 5]. In approximately half of the patients, KOT is asymptomatic while in others, pain, swelling, drainage, tooth mobility or displacement and bone expansion can be seen [ 4, 6]. The recurrence of KOT varies 5-62% [ 7]. In radiograph images, KOT is a uni- or multi-locular well-defined radiolucent cyst, bounded by smooth or scalloped margins and sclerotic borders [ 5]. Association with at least one unerupted tooth can be seen in 30% of the cases, especially in younger patients [ 8].

In histological examination, KOTs are characterized by a fibrous wall, lined with corrugated parakeratinized stratified squamous epithelium of 6-10 cell layers in thickness without rete pegs, which makes a flat interface between the epithelium and the connective tissue [ 6- 7, 9]. The well-defined basal layer contains palisaded columnar or cuboidal cells with higher mitotic activity than other dentigerous cysts [ 8]. Although the KOT treatment remains controversial, it is commonly classified as either conservative or aggressive. The conservative treatment involves “simple enucleation, with or without curettage, or marsupialization”, whereas the aggressive one involves chemical curettage, peripheral ostectomy, and resection. The treatment of choice should be based on the lowest risk of recurrence and morbidity [ 4]. 

## Case Presentation

A 9-year-old boy referred to the department of oral and maxillofacial surgery of Rajaei Acute Care Surgical Hospital affiliated to Shiraz University of Medical Sciences with a chief complaint of swelling in the right body of mandible. The history of papilloma in the dorsal region of the tongue and familial multiple KOTs were reported. Extra oral examination revealed a non-tender, firm swelling on the same side. Intra oral examination revealed a painless, firm swelling in the buccal vestibule, extending from distal aspect of canine to the mesial aspect of the first molar. No systemic pain was observed and the patient’s general condition was good.

**Figure 1:**
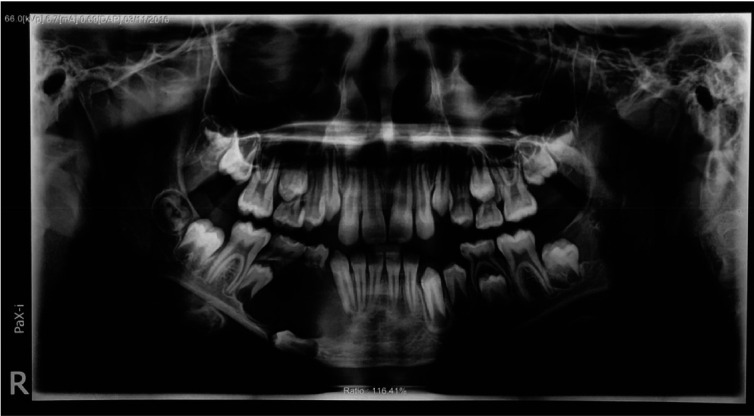
Panoramic image shows the lesion site pre-operatively

The panoramic image showed a multilocular well-defined radiolucent lesion in the right side of mandible from the right canine to mesial of the non-erupted second premolar. The lesion had expanded from alveolar crest to inferior border of the mandible, and there were some septa inside. Displacement of the first premolar and root resorption of the primary first molar were observed (Figure 1).

**Figure 2:**
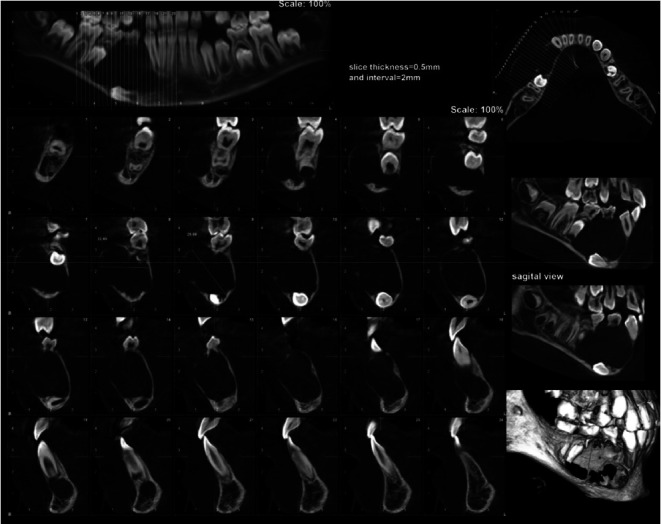
Cross-sectional view of CBCT (cone beam computed tomography) shows expansion of the lesion, which causes displacement and impaction of permanent first premolar into inferior border of the mandible

**Figure 3:**
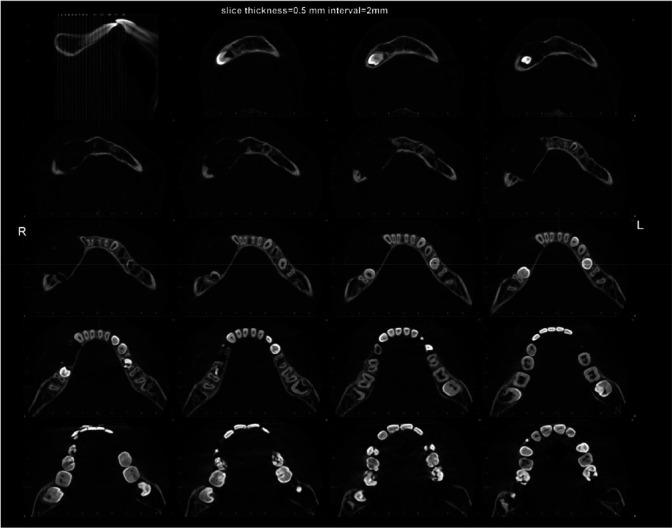
Axial view of CBCT shows thinning and expansion of both buccal and lingual cortical plates at the lesion site

The cone beam computed tomography (CBCT) images showed a large multilocular expansile radiolucent lesion in the right side of mandible that had caused displacement and impaction of permanent first premolar, root divergence of canine and second premolar as well as root resorption of the primary first molar. Loss of continuity of cortical borders of mandibular canal at the affected site was seen. Thinning and expansion of mandibular buccal and lingual cortical plates as well as perforation of buccal mandibular cortex were observed (Figures 2 and 3).

There was another expansile lytic lesion in the left mandibular molar area, causing destruction or failure to develop tooth bud of the left third molar.

Based on clinical and radiographic findings, the differential diagnosis was compatible with dentigerous cyst and then KOT. The treatment possibilities were discussed with his parents. 

**Figure 4:**
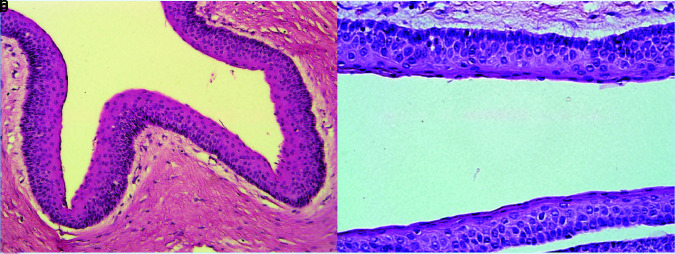
A. The connective tissue shows mild infiltration of inflammatory cells. B. Histologic examination showing the parakeratotic uniform stratified squamous epithelium with hyperchromic palisaded basal layer. (Magnification: Panel A: 200×, Panel B: 400×)

For histopathologic findings, two incisional biopsies were initially performed to approve the diagnosis. Microscopic examination showed a cystic lesion lined by corrugated parakeratotic uniform stratified squamous epithelium with hyperchromic palisaded basal layer, and the connective tissue revealed mild infiltration of inflammatory cells (Figure 4).

Treatment started with prep and drape under general and hypotension anesthesia in supine position. First, right sulcular incision was made and musculomucosal flap was reflected. Inferior alveolar nerve was explored and preserved. Next, marsupialization of pathologic lesion was done and obturator was applied. Afterward, mucoperiosteal flap was replaced and sutured with vicryl 3/0. Another sulcular incision in the left side of the mandible was made and mucoperiosteal flap was reflected. Then, unerupted tooth No. 7 was extracted and pathologic lesion in this site was excised. Mucoperiosteal flap was replaced and sutured with vicryl 3/0. After discharging from hospital, the patient returned periodically for the follow-up. 

**Figure 5:**
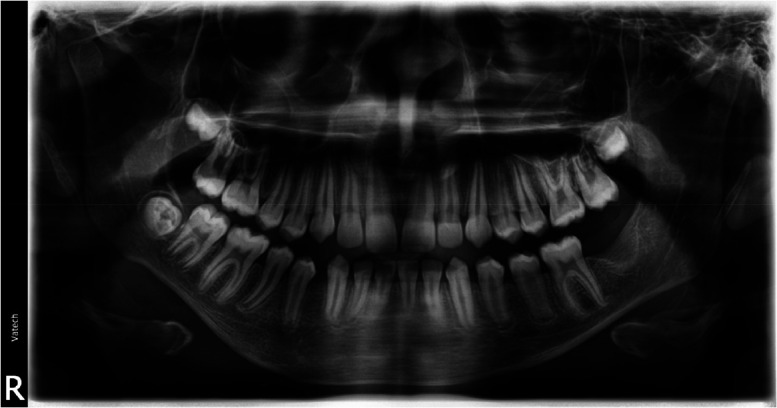
Panoramic image shows the lesion site after two years of follow-up

After one year, the lesion size had decreased and the first mandibular premolar had completely erupted. After two years, the panoramic radiograph showed adequate bone healing and no evidence of lesion recurrence was observed (Figure 5). The first mandibular premolar showed normal morphology and normal function with pulp vitality.

## Discussion

Various approaches are utilized to treat KOTs. The conservative treatments for KOTs are marsupialization (possibly followed by residual cystectomy) and decompression. The invasive treatments are resection, peripheral osteotomy, and enucleation (possibly followed by chemical agents like Corney’s solution). Due to high recurrence of KOTs, the best treatment plan is still controversial [ 5, 10]. It has been noted that treatment of choice should be based on the lowest risk of recurrence and morbidity [ 4]. Pogrel [ 11] concluded that decompression or marsupialization can preserve more vital structures and has the same success rate as the more aggressive treatments. Marsupialization is to convert the cyst into a pouch. Mandibular cysts are marsupialized into the oral cavity while maxillary cysts can be exposed into the maxillary sinus and nasal cavity as well as oral cavity [ 11]. In another study by Pogrel [ 12], it was discussed that by marsupialization, parakeratinized KOTs might get resolved completely and the teeth within the cyst can become upright and erupt. However, the recurrence rate should not be the only factor when choosing a treatment plan. In preadolescents, it is possible that the associated teeth would erupt spontaneously after marsupialization, according to the age and depth of the associated teeth [ 13]. According to Oh *et al*. [ 14], marsupialization led to obvious change in epithelium, which was no longer similar to the appearance of KOT. Their study also proved that “bone formation was significantly enhanced in the KOT capsule wall adjacent to bone after marsupialization.”

A recent study [ 15] evaluated 34 KOTs in 27 patients who had undergone decompression followed by enucleation. By this technique, the average maximum diameter of KOT s had decreased; moreover, the thin and brittle cyst wall of the KOTs had thickened.

The authors [ 15] claimed that decompression as an efficient treatment of KOTs can reduce its recurrence rate. It was also mentioned that due to a few differences between marsupialization and decompression, the term marsupialization was unified with decompression.

A written informed consent was obtained from patient's parents.

## Conclusion

In our case study, marsupialization was found to be an effective treatment in inducing the eruption of mandibular premolar associated with KOT in preadolescent. It seems that this treatment modality can be a reliable procedure to reduce recurrence tendency of KOT.
